# Inhibition of visceral adipose tissue-derived pathogenic signals by activation of adenosine A_2A_R improves hepatic and cardiac dysfunction of NASH mice

**DOI:** 10.1152/ajpgi.00104.2023

**Published:** 2024-01-22

**Authors:** Chia-Chang Huang, Hsiao-Yun Yeh, Roger Lin, Tsai-Ling Liao, Hsiao-Chin Shen, Ying-Ying Yang, Han-Chieh Lin

**Affiliations:** ^1^Department of Medical Education, Taipei Veterans General Hospital, Taipei, Taiwan; ^2^Division of Gastroenterology and Hepatology of Department of Internal Medicine, Taipei Veterans General Hospital, Taipei, Taiwan; ^3^Department of Internal Medicine, Taipei Veterans General Hospital, Taipei, Taiwan; ^4^Institute of Clinical Medicine, Taipei Veterans General Hospital, Taipei, Taiwan; ^5^Faculty of Medicine, School of Medicine, National Yang-Ming Chiao Tung University, Taipei, Taiwan; ^6^Department of Medical Research, Taichung Veterans General Hospital, Taichung, Taiwan

**Keywords:** A2AR, hepatic and cardiac dysfunction, nonalcoholic steatohepatitis, steatosis, visceral adipose tissue

## Abstract

A_2A_R-disrupted mice is characterized by severe systemic and visceral adipose tissue (VAT) inflammation. Increasing adenosine cyclase (AC), cAMP, and protein kinase A (PKA) formation through A_2A_R activation suppress systemic/VAT inflammation in obese mice. This study explores the effects of 4 wk A_2A_R agonist PSB0777 treatment on the VAT-driven pathogenic signals in hepatic and cardiac dysfunction of nonalcoholic steatohepatitis (NASH) obese mice. Among NASH mice with cardiac dysfunction, simultaneous decrease in the A_2A_R, AC, cAMP, and PKA levels were observed in VAT, liver, and heart. PSB0777 treatment significantly restores AC, cAMP, PKA, and hormone-sensitive lipase (HSL) levels, decreased *SREBP-1/FASN*, MCP-1, and CD68 levels, reduces infiltrated CD11b^+ ^F4/80^+^ cells and adipogenesis in VAT of NASH + PSB0777 mice. The changes in VAT were accompanied by the suppression of hepatic and cardiac lipogenic/inflammatory/injury/apoptotic/fibrotic markers, the normalization of cardiac contractile [sarco/endoplasmic reticulum Ca^2+ ^ATPase (SERCA2)] marker, and cardiac dysfunction. The in vitro approach revealed that conditioned media (CM) of VAT of NASH mice (CMnash) trigger palmitic acid (PA)-like lipotoxic (lipogenic/inflammatory/apoptotic/fibrotic) effects in AML-12 and H9c2 cell systems. Significantly, A_2A_R agonist pretreatment-related normalization of A_2A_R-AC-cAMP-PKA levels was associated with the attenuation of CMnash-related upregulation of lipotoxic markers and the normalization of lipolytic (AML-12 cells) or contractile (H9C2 cells) marker/contraction. The in vivo and in vitro experiments revealed that A_2A_R agonists are potential agent to inhibit the effects of VAT inflammation-driven pathogenic signals on the hepatic and cardiac lipogenesis, inflammation, injury, apoptosis, fibrosis, hypocontractility, and subsequently improve hepatic and cardiac dysfunction in NASH mice.

**NEW & NOTEWORTHY** Protective role of adenosine A_2A_R receptor (A_2A_R) and AC-cAMP-PKA signaling against nonalcoholic fatty liver disease (NAFLD)/nonalcoholic steatohepatitis (NASH) possibly via its actions on adipocytes is well known in the past decade. Thus, this study evaluates pharmacological activities of A_2A_R agonist PSB0777, which has already demonstrated to treat NASH. In this study, the inhibition of visceral adipose tissue-derived pathogenic signals by activation of adenosine A_2A_R with A_2A_R agonist PSB0777 improves the hepatic and cardiac dysfunction of high-fat diet (HFD)-induced NASH mice.

## Graphical abstract

**Figure:**
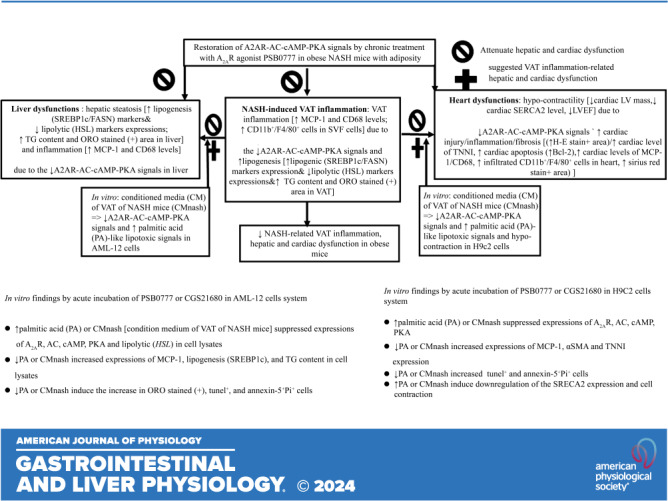

## INTRODUCTION

The prevalence of nonalcoholic fatty liver disease (NAFLD), nonalcoholic steatohepatitis (NASH), hepatic fibrosis, and cirrhosis are increasing worldwide (1). Patients with NAFLD had high frequency of advanced heart failure (2, 3). NAFLD is present in a large portion of congestive heart failure patients with severe left ventricle fibrosis (2, 3).

Increased visceral adiposity has been reported as a major contributor of hepatic steatosis and fibrosis in NAFLD (4). Hepatic steatosis and fibrosis are associated with progressive cardiac dysfunction (5). Patients with NAFLD are characterized by simultaneous cardiac structure change and cardiac dysfunction compared with non-NAFLD patients (6, 7). Low-grade systemic and visceral adipose tissue (VAT) inflammations are involved in the NAFLD/NASH disease progression and development of cardiac dysfunction (8, 9). Increased histologic severity of NAFLD was associated with more severe cardiac dysfunction, which suggests synergistic pathogenic mechanisms of hepatic inflammation and fibrosis, and cardiac dysfunction (10).

Adenosine A_2A_R receptor, the superfamily of G protein-coupled receptor, exerts powerful anti-inflammatory effects in adipose, hepatic, and cardiac tissue (11–14). Treatment of high-fat diet-fed C57BL/6 mice with the A_2A_R receptor agonist (CGS21680 or PSB0777) attenuated the severity of systemic adiposity (15). In adipose tissue, the elevated cAMP level triggers protein kinase A (PKA), which is the downstream of A_2A_R (16). cAMP-dependent PKA activation attenuates SREBP-1 activity and SREBP-1-mediated lipogenesis (17, 18). Activation of PKA signals in turn upregulates the hormone-sensitive lipase (*hosl-1)* expression, which mediated fat mobilization and lipolysis in adipose tissue (19).

Systemic A_2A_R-disrupted mice are characterized by increased severity of high-fat diet-induced systemic and hepatic inflammation. Disruption of A_2A_R in hepatocytes accounts for the increased severity of NAFLD likely through increasing inflammation and through elevating lipogenic events due to stimulation of SREBP1c expression and transcription activity (14). Administration of 2 wk of the A_2A_R agonist CGS21680 suppressed systemic and adipose tissue inflammation in HFD-fed obese mice (13, 20). Activators of A_2A_R activate adenosine cyclase (AC), a membrane effector synthesizing cAMP from ATP, to increase the formation of cAMP (21).

A_2A_R mRNA level is decreased in cardiac tissue of patients with heart failure (22). A_2A_R is abundant in cardiomyocytes and stimulation of cardiac A_2A_R protect the heart from reperfusion cardiac damage by improving energy metabolism (increase ATP content; 23). Activation of A_2A_R and its downstream cAMP-PKA can enhance cardiac contractility in an experimental model of ischemia-reperfusion injury (24). Hepatic triglyceride content was associated with a reduction of the myocardial ATP level, which represented impaired cardiac energy metabolism in patients with type 2 diabetes (25).

This study aims to explore the role of A_2A_R-AC-cAMP-PKA signals in the adipose tissue on its lipogenesis and inflammation, as well as corresponding hepatic and cardiac inflammation, and cardiac dysfunction of diet-induced NASH mice. Furthermore, the effects of the chronic A_2A_R agonist PSB0777 treatment on the abovementioned adipose tissue, liver, and cardiac dysfunction were evaluated. Finally, in vitro experiments, the cross talk between primary isolated mice adipose tissue and hepatocytes and cardiomyocytes were explored.

## MATERIALS AND METHODS

### Animals

All animal experiments complied with the ARRIVE guidelines and were carried out in accordance with National Institutes of Health guidelines for animal welfare and were conducted with the Animal Research Committee of Yang-Ming Chiao Tung University with approval Nos. of 1110429 and 1110318. Twelve-week-old male C57BL/6 mice were bought from Jackson Laboratories, Bar Harbor, ME. Mice were housed in the specific pathogen-free (SPF) facility and adapted to the new conditions for 2 days before the experiment. Mice were maintained under a temperature-controlled (22°C–23°C) room with a 12-h light/dark cycle. At the end of the experiments, the mice were euthanized with 2–3 times the anesthetic dose of zoletil. All efforts were made to minimize animal numbers necessary to produce reliable results and suffering was reduced by administering anesthetics (zoletil and xylocaine).

Twelve-week-old C57BL/6 mice (Jackson Laboratories, Bar Harbor, ME) were fed with 24-wk normal chow (NC, Laboratory Autoclavable Rodent Diet 5010) as control (Ctrl) group or a NASH group receiving 24-wk high-fat diet (HFD), HFD (D12492, Research Diet, Inc., Brunswick, NJ), which consisted of 37% fat and 5.24 kcal/g. Male C57BL/6 mice were weighed every week, an average weight >40 g was set as the criterion for initiating the streptozotocin (STZ) injection. To prepare the injection reagent, STZ was dissolved in normal saline, and 0.1 M sodium citrate (pH= 4.5) was used as the buffer solution. Three doses of STZ (40 mg/kg/day) were injected intraperitoneally at the 3, 5, and 7 days of the 20th week after HFD feeding. Streptozotocin-high-fat diet (HFD)-created NASH mice were used in the following experiments.

**Figure 1.**
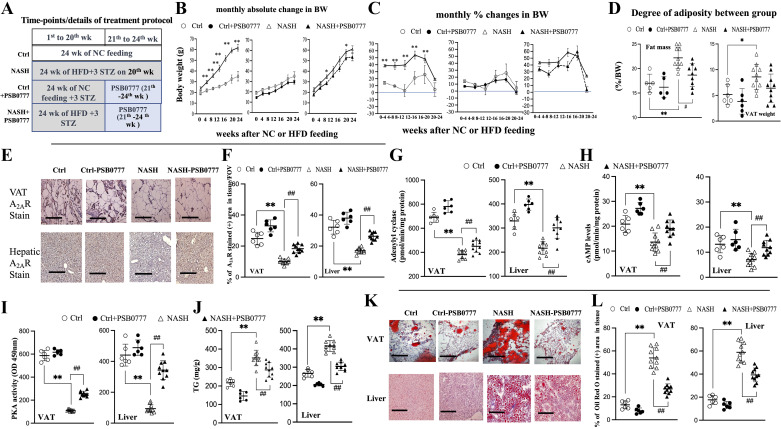
Chronic A_2A_R agonist PSB0777 treatment suppresses adiposity and lipotoxic signals in visceral adipose tissue (VAT) and livers of HFD-induced male NASH mice. Treatment protocol (*A*); curves of monthly absolute changes (*B*), and percent changes (%) (*C*) of body weight (BW) after NC or HFD feeding; adiposity (increased fat mass/VAT weight) between groups (*D*); representative image (×200, scale bar 100 μm; *E*) and scatter dot plot (*F*) of the A_2A_R IHC stained (+) area in VAT and liver; levels of adenylyl cyclase (*G*), and cAMP (*H*), PKA (protein kinase A; *I*), and TG contents (*J*) in VAT and liver; representative image (×200, scale bar 100 μm; *K*) and scatter dot plot of Oil red O (ORO) stained (+) area (*L*) in VAT and liver. *,**P < 0.05, 0.01 vs. Ctrl group (*n* = 7); ##*P* < 0.01 vs. NASH group (*n* = 10). Figures are prepared by GraphPad Prism 6.0 310 (GraphPad Software, La Jolla, CA) and *t* test between PA/CMnash vs. PA+PSB0777/CMnash+PSB0777 or PA/CMnash vs. or PA+CGS21680/CMnash+PSB0777 were performed with SPSS 19.0 (SPSS, Inc., Chicago, IL). Figures are prepared by GraphPad Prism 6.0 310 (GraphPad Software, La Jolla, CA) and one-way ANOVA with post hoc Tukey’s multiple comparisons tests were performed with SPSS 19.0 (SPSS, Inc., Chicago, IL). HFD, high-fat diet; IHC, immunohistochemical; NASH, nonalcoholic steatohepatitis; NC, normal chow; TG, triglyceride.

PSB0777, a novel highly polar A_2A_R-selective agonist, is stable in an acid environment and devoid of cardiovascular adverse effects of other A_2A_R agonists. It had been reported that this dose of chronic PSB0777 treatment ameliorate NASH and its complications in NASH animals (15). PSB0777 (0.4 mg/kg/day) was dissolved in DMSO at a concentration of 20 mM, stock solution was used to spike three tubes containing PSB (cutoff concentration 200 μM) with a final solvent content of 1% DMSO and suspended in 1% methocel (a water-soluble polymers used as a carrier of drug) for ALZET osmotic pump-based administration (26). The intraperitoneal placement of 28 days ALZET osmotic pump in mice was undergone at the beginning of 21st week for drug releasing from the 21st to 24th week after HFD or NC feeding. Then, the osmotic pump filled with DMSO + methocel alone or osmotic pump filled with DMSO + methocel+PSB0777 were intraperitoneal placement in Ctrl/NASH and NASH + PSB0777/Ctrl + PSB0777 groups, separately. This created experimental groups, namely Ctrl (*n* = 7), NASH (*n* = 10), Ctrl + PSB0777 (*n* = 7), and NASH + PSB0777 (*n* = 10) mice (Fig. 1*A*).

### Experimental Design

Among the Ctrl, NASH, Ctrl + PSB0777, NASH + PSB0777 mice, cardiac echogram, and body composition were measured. Then, liver tissue, visceral adipose tissue (VAT; pericardial fat, bilateral epididymal fat pad, bilateral peri-renal fat, and mesenteric adipose tissues), and cardiac tissue of different groups of mice were collected and weighed after euthanasia.

### Body Composition

This was determined by SkyScan 1076 computed tomography (Bruker microCT, Belgium) scanner of Taiwan mouse clinics. Body composition (fat and lean mass) was measured three times in each mice and results are presented as means of these measurements and are expressed as a percentage of total body weight.

### Echocardiography

To determine the effects of chronic A_2A_R agonist PSB0777 treatment on NASH-associated cardiac dysfunction in vivo, the cardiac morphology and function were assessed by M-mode transthoracic echocardiography using a high-frequency and high-resolution echocardiography system (Vevo 3100, FUJIFILM VisualSonics, Inc., Toronto, Canada) equipped with a 15- and 40-MHz ultrasound probe (Phillips Sonos 5500 System) in mice lightly anesthetized with inhaled 2% isoflurane in oxygen. Prewarmed echo gel is applied, and the echocardiography probe is positioned. The images were collected in the short and long axes; the data represent the averaged values of 3–5 cardiac cycles. Diastolic and systolic volumes were acquired by applying Simpson’s rule of disks to the serially acquired short-axis images. Fractional shortening was calculated as: FS = (LVDd − LVDs)/LVDd × 100%, where LVDd is left ventricular diastolic dimension and LVDs is left ventricular systolic dimension. Left ventricular diameters during diastole (LVIDd), left ventricular diameter during systole (LVIDs), and heart rate (HR) were determined from long-axis M-modes. Left ventricular ejection fraction (LVEF) was calculated as: LVEF = (LVEDV − LVESV)/LVEDV × 100%, where LVEDV is left ventricular end-diastolic volume and LVESV is left ventricular end-systolic volume. Relative wall thickness was calculated as: (diastolic posterior wall thickness + diastolic anterior wall thickness)/LVIDd. An experienced cardiologist and an echocardiography expert blinded to experimental groups performed all measurements.

### Serum, Tissue, and Cellular Levels of Adenylyl Cyclase, cAMP, PKA, and Various Pathogenic Markers

Expressions of various pathogenic markers in visceral adipose tissues (VAT), liver, AML-12, heart, and H9C2 cells were measured by Western blot, RT-PCR and adenylyl cyclase (AC, pmol/min/mg protein), cAMP (pmol/mg), and PKA (OD, 540 nM) activities ELISA kits (27). For Western blot analysis, lipids in VAT were eliminated via centrifugation of the solubilized samples at 13,000 rpm for 30 min at 4°C. Heart and liver homogenates were fractionated based on their detergent solubility and centrifugation, as previously described, to produce enriched fractions. Cells were homogenized in a lysis buffer supplemented with protease inhibitors (1 mm phenylmethylsulfonyl fluoride; 100 mm; 2 g/mL aprotinin, 2 g/mL leupeptin). The soluble fraction represents the cytosol. The fraction soluble in a 1% Triton X-100 buffer represents the membranes. The final fraction, soluble in 2% SDS (SDS-PAGE sample buffer) represents the myofilaments/nuclei. Fractionation was confirmed using compartment-specific antibodies. The supernatant was collected, and the protein concentration was checked by Bradford assay at 595 nm. Protein concentrations in tissues/cell lysates were determined by the Pierce BCA protein assay kit with Bradford reagent (Thermo Fisher Scientific, Inc., IL). Tissue homogenates or cell lysate were added at 0°C to 10 mL of variables in 23 assay mix [1 mM ATP, 20 mM DTT, 0.4 mM IBMX, and 4 mM of either MgCl_2_ (Mg21 assay mix) or MnCl2 (Mn21 assay mix) in lysis buffer (LB)]. Reactions were initiated by transferring the samples at 23°C water bath and were terminated by addition of 10 mL 0.4 M EDTA and by boiling the samples for 1 min. The appropriate primary [A_2_AR, CD68, MCP-1, SERCA2 (Santa Cruz, Biotechnology, Inc.), phospho-(Ser/Thr) PKA, Bcl-2 (Cell Signaling Technology), troponin-I (TNNI), GADPH (Abcam, Cambridge, UK)] and secondary antibody were applied to each polyvinylidene fluoride membranes. Image J software was used to assess the density of the protein of the interest band, which was then normalized to the GADPH protein band. For q-RT-PCR, total RNA was purified from tissues/cell lysates using TRIzol reagent (Invitrogen) and an RNeasy mini kit (Qiagen). The quantity and quality of the RNA were determined spectroscopically using a nanodrop (Thermo Scientific). Total RNA was used to synthesize cDNA using the Transcriptor cDNA first-strand synthesis kit (Roche) according to the manufacturer’s protocol and was resuspended in 50 λ of H_2_O. cDNA samples (2 λ) were used for real-time PCR in a total volume of 25 λ using SYBR green reagent (Invitrogen) and specific primers (Table 1) on a qPCR machine (Applied Biosystems 7300 Sequence Detection System). All real-time PCRs were performed in duplicates. Data were generated and analyzed using SDS 2.3 and RQ manager 1.2 software. The samples’ threshold cycle (Ct) values were computed, a script levels were determined using the 2^−ΔCt^ method, and the expression values were normalized to the level of 18S mRNA. Serum MCP-1, ALT, and myocardial injury markers (CK and cTnI) were measured.

**Table 1.** Primers of various genes used in this study

Gene Name	Forwards	Reverse
*ADORA2A*	5′-CCGAATTCCACTCCGGTACA-3′	5′-CAGTTGTTCCAGCCCAGCAT-3′
*HSL*	5′-GGCTTACTGGGCACAGATACCT-3′	5′-CTGAAGGCTCTGAGTTGCTCAA-3′
*SREBP1*c	5′-ATCGGCGCGGAAGCTGTCGGGGTAGCGTC-3′	5′-ACTGTCTTGGTTGTTGATGAGCTGGAGCAT-3′
*FASN*	5′-GCTGCGGAAACTTCAGGAAAT-3′	5′-AGAGACGTGTCACTCCTGGACTT-3′
*CD68*	5′-GACCTACATCAGAGCCCGAGT-3′	5′-CGCCATGAATGTCCACTG-3′
*Bcl-2*	5′-GAGTACCTGAACCGGCATCTG-3′	5-CTTCAGAGACAGCCAGGAGAAA-3′
αSMA	5′-CGGGCTTTGCTGGTGATG -3′	5′-CCCTCGATGGATGGGAAA-3′
*SERCA2*	5′-TCGACAGGACAGAAAGAGTGTG-3′	5′AAACTGAATTCAACTCACCAGC-3′
*18S*	5′-GCGAAAGCATTTGCCAGAA-3′	5′-GGCATCGTTTATGGTCGGAAC-3′

*ADORA2A*, gene of adenosine A_2A_ receptor; FASN, gene of fatty acid synthase; *HSL*, gene of hormone-sensitive lipase; *SREBP1c*, gene of sterol regulatory element-binding proteins-1c.

### Hematoxylin and Eosin, Immunochemistrical, and Oil-Red O Staining

Fresh mouse VAT, liver, and heart were fixed in 10% neutral formalin, embedded in paraffin, and sectioned in 4-μm thickness. Hematoxylin and eosin (H&E) staining was performed for estimation of the degree of mononuclear cells infiltration, which was classified as mild (involvement < 25% of the tissue), moderate (25%–50% of the tissue), or severe (involvement > 50% of the tissue). The extent of heart (interstitial) fibrosis was visualized after Sirius red staining for calculating collagen deposition in the total LV dimension (Sigma-Aldrich, St. Louis, MO). For immunohistochemical (IHC) assays, slides were deparaffinized and rehydrated with ethanol, then sequentially incubated with 0.3% H_2_O_2_ to block endogenous peroxidase activity. Next, the slides were incubated with various primary antibodies including A_2_AR and MCP-1 for 2 h at room temperature. Subsequently, a biotinylated secondary antibody and avidin-biotin complex reagent were added, and color development was induced by DAB and visualized under a light microscope for IHC. After being air-dried at room temperature, the slides were fixed in ice-cold formalin for 10 min and rinsed immediately in distilled water three times. After being air-dried again, the slides were stained for 10–15 min with freshly prepared oil-red O (ORO) working solution (Sigma-Aldrich, MO) and rinsed with 60% isopropanol. After being stained in Mayer’s hematoxylin for 30–60 s, the slides were washed thoroughly in distilled water, mounted with glycerin jelly, and photographed under an electron microscope (Olympus, Japan). Intracellular lipid accumulation was detected using ORO staining in tissue and AML-12 cell layer after rinsing with phosphate-buffered saline (PBS) twice, fixed in 10% buffered formalin for 10 min at room temperature, incubated with 60% isopropanol for 20 min, and washed with 60% isopropanol to remove background staining and finally rinsed with PBS. The stained area/cells were observed by microscopy.

### Triglyceride Content in Liver, VAT of Mice and AML-12 Cells

Triglycerides (mg/g wet tissue or mmol/g protein) in mice liver/VAT and AML-12 cells were measured using commercial kits. In brief, 100 mg of tissues/cells (10e^8^) were homogenized in 2 mL chloroform/methanol (2/1); the mixture was then agitated in an orbital shaker at room temperature for 20 min and centrifuged. The lower phase, which contained most of the tissue lipids, was subsequently recovered, and the solvent was washed with 400 μL of 0.9% NaCl solution. The mixture was centrifuged at low speed to separate the two phases. The lower, chloroform phase containing the lipids was recovered and evaporated under vacuum. The recovered lipids were mixed with 10% Triton X-100 in 11 isopropanol.

### Macrophage Infiltration in VAT and Heart

For flow cytometry analysis, mice VAT and heart (10 mg) were minced and digested for 45 min at 37°C with type 1 collagenase (4 mg/mL) in Dulbecco’s modified Eagle’s medium (DMEM) containing 5% fetal bovine serum (FBS) (pH 7.4). After the addition of 3 vol of PBS containing 5% FBS, the digested tissue was filtered through a nylon mesh (100 μm). The red blood cells present in the stromal vascular fraction (SVF) of VAT and heart pellet were lysed with Pharm Lyse (BD Biosciences), and the resulting cell suspensions (3 × 10^6^ cells/g tissue) were fractionated on 30/60% Percoll gradients at 1,000 *g* for 25 min, and mononuclear cells (MCs) were collected from the interface. Because of the low number of MC obtained, cells from 3 to 10 mice/experiment were pooled. NC was suspended in PBS containing 2 mM EDTA. Cell viability was assessed by incubation in the amine-reactive dye Aqua [(Invitrogen) (1:500) dilution in Ca^2+^ and Mg^2+^ free PBS] for 30 min in the dark at room temperature (RT), followed by a single wash in 1× PBS, and dead cells per debris were excluded. Then, freshly prepared NC suspensions (3 × 10^5^ cells in 100 µL total volume) were exposed to FcR-blocking reagent (BD Biosciences, San Jose, CA) for 20 min before labeling for 1 h at 4°C with fluorescently labeled antibodies and washed in FACS buffer. Primary antibodies were: allophycocyanin (APC)-conjugated/PE-conjugated anti-F4/80 (Thermo Fisher Scientific), FITC-conjugated CD11b antibodies (Thermo Fisher Scientific) at dilutions following manufacturer recommendations. F4/80-positive cells were gated out from the live cells, and we used CD11b to confirm the infiltrated macrophage in tissue. Total cell counts were estimated by the total number of events per samples per milligram of tissue. All data collection from 10^4^ events were acquired, including forward scatter (FSC), side scatter (SSC), and multiple fluorescence channels detecting fluorescein isothiocyanate (FITC), allophycocyanin (APC), and phycoerythrin (PE) were analyzed with flow cytometer’s software (Supplemental Materials and Methods). The results are presented as a percentage (%) of CD11b + F4/80+ cells among CD11b+ positive cells (/gram tissue). For dead cells per debris exclusion, FSC and SSC measurements give an estimation of the size and granularity of the cells and are most useful to find viable, single-cell events (28). Because of morphology change (blebbing), dead cells can be distinguishable from normal cells for the reduced cell size (low FSC) and enhanced density (high SSC) typically found at the bottom left corner of the FSC/SSC dot plot. It has been reported that avoiding drawing a gate of low FSC and high SSC can exclude dead cells/debris from the analysis (29). Annexin staining is good for detecting cells in dead cells. Previous study had reported that cells in the low FSC/high SSC subpopulation were all found to be annexin V-positive, whereas the cells with the normal FSC/SSC pattern were annexin V-negative (30).

### Preparation of Conditioned Media from VAT of NASH Mice

VAT from male NASH mice was minced into 2- to 3-mm^3^ fragments and incubated at 100 ± 5 mg/mL in Dulbecco’s modified Eagle medium (DMEM) containing 4.5 g/L glucose, 10% fetal bovine serum (FBS), 50 U/mL penicillin, 50 μg/mL streptomycin, and 4 mM glutamine (Biological industries, Beit HaEmek, Israel) for 24 h. Then, explants were washed and reincubated in DMEM containing 0.1% FBS for an additional 24 h. These media NASH mice (CMnash) were collected and kept at −80°C until use.

### Effects of A_2A_R Agonist on the Palmitic Acid-like Lipotoxic Effects Induced by CMnash in Cell Lysates of AML-12 and H9c2 Cells

Mouse α liver cells (AML-12) and H9c2 cardiomyocyte cell line were cultured in Dulbecco’s modified Eagle’s medium (DMEM) containing 4.5 g/L glucose and 10% FBS in an atmosphere of 95% air and 5% CO_2_ at 37°C. Accumulating evidence suggests that metabolically hazardous visceral adipose tissue (VAT) contributes to NASH progression by releasing saturated fatty acids (SFA) and proinflammatory mediators (31, 32). Palmitic acid (PA), an abundant SFA, which are powerful at inducing steatosis and inflammation, and is usually elevated in obesity and NASH (33). So, the in vitro approach aims to evaluate whether CMnash trigger PA-like lipotoxic effects, including adipogenesis, inflammation, injury, fibrosis, apoptosis, and hypo-\contraction, using AML-12 and H9c2 cell line as model systems. Meanwhile, the effects of A_2A_R agonist on the lipotoxic signals induced by CMnash and PA.

Various in vitro experiments were undergone to assess the effects of A_2A_R agonist (PSB0777 or CGS21680, 20 nM) by coincubation of 24 h on the palmitic acid (PA) (0.2 mM) or CMnash-induced pathogenic (adipogenic, inflammatory, injury, fibrotic, apoptotic, and hypocontractility) markers. In preliminary experiments, AML-12 and H9c2 cells were coincubated with PSB0777 or CGS21680 to reverse the PA-suppressed A_2A_R expression levels, and 20 nM of PSB0777/CGS21680 caused the best suppression that cannot further enhanced by higher concentration. Then, cAMP (pmol/mg protein), adenylyl cyclase (pmol/min/mg protein), PKA activities/levels, mRNA and proteins expressions, lipogenesis (Red Oil O staining), and apoptosis (annexin-5+/Pi+, and tunel+) were measured in the cell lysates of AML-12 and H9c2 cells with PA alone or combined with either PSB0777 or CGS21680.

### Effects of A_2A_R Agonist on the CMnash-Induced Palmitic Acid-like Effects on the Contraction of H9c2 Cells

H9c2 cells contraction was measured by changes in planar surface area by culturing them in 6-mm flat bottom plates ∼5 × 10^5^ cells/plate. Culture plates were then mounted on the heated stage of an inverted light microscope for the contractility assay. Changes in the planar surface areas in response to treatment were observed using a video camera, and images of the cells were captured serially at 16, 24, and 48 h. The perimeters of individual cells with clearly defined borders were outlined, and the planar surface area was calculated as a percentage change from baseline. All data were calculated using WIPL ∼ b software (Foreseen Science and Technology, Tainan, Taiwan). Changes in planar surface areas of cells in response to treatment are compared with buffer group.

### Statistical Analysis

Data are presented as means and standard errors. The statistical significance of differences between study groups was evaluated using *t* test and one-way ANOVA with post hoc Tukey’s multiple comparisons test using SPSS 19.0 (SPSS, Inc., Chicago, IL). All graphs were produced with Prism software 6.0 (GraphPad Software, La Jolla, CA). A *P* value ≤ 0.05 is considered statistically significant.

### Materials

A_2A_R, PKA, CD68, MCP-1, Bcl-2, SERCA2, troponin-I (TNNI), FITC-conjugated F4/80, and PE-conjugated CD11b, and GADPH antibodies were purchased from Cell Signaling Technology (Danvers, MA), Abcam (Cambridge, MA), and Santa Cruz Biotechnology, Inc. (Santa Cruz, CA). PSB0777/CGS21680 were purchased from TOCRIS Bioscience (R&D). Mouse adenylate cyclase ELISA kit was purchased from MyBioSource, Inc. (San Diego, CA; detection range: 5.0–100 ng/mL, sensitivity: 1.0 ng/mL) and mouse cAMP ELISA kit was purchased from Cayman Chemical (detection range: 0.078–10 pmol/mL, sensitivity: 0.1 pmol/mL). PKA activity (OD, 450 nm) and mouse PKA kinase assay kit (Abcam, Inc., Cambridge, MA), TUNEL assay (Abcam, Inc., Cambridge, MA), and Annexin V Apoptosis detection kits (Elabscience, Biotechnology) were used for measuring of tissue and cellular activities/levels, and triglyceride assay kits were purchased from Sigma-Aldrich (St. Louis, MO). PCR primers and related substances were prepared using a DNA synthesizer (Protech Technology, Solon, OH). Other substances were purchased from Sigma Chemical Co. (St. Louis, MO).

## RESULTS

### Systemic and Paralleled Antilipogenesis Effects of Chronic PSB0777 Treatment in VAT and Liver of NASH Mice

**Figure 2.**
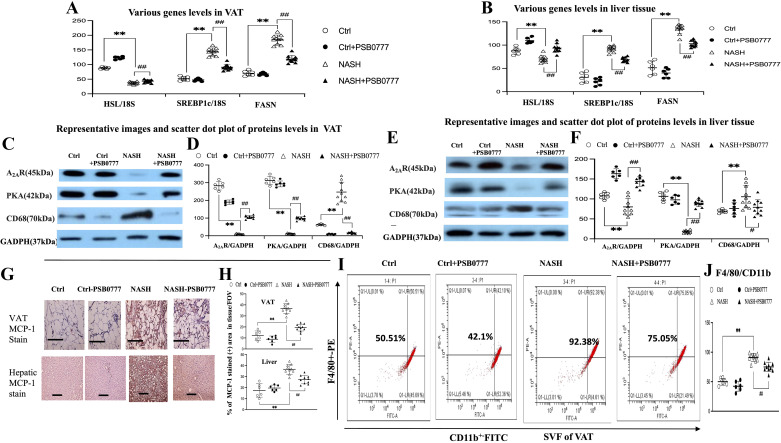
The A_2A_R agonist PSB0777 treatment suppressed inflammation and lipogenesis simultaneously in visceral adipose tissue (VAT) and liver of male NASH mice. Relative mRNA levels (/18S) of HSL, SREBP1c, and FASN in VAT and livers (*A* and *B*); representative image (×200, scale bar 100 μm) and scatter dot plot of protein levels of A_2A_R, PKA, CD68, and MCP-1 levels in VAT and livers (*C–H*); representative images and scatter dot plot graph of flow cytometry assessed degree of infiltration of CD11b+F4/80+ cells in stromal vascular fraction (SVF) cells of VAT (*I* and *J*). **P < 0.01 vs. Ctrl group (*n* = 7); #,##*P* < 0.05, 0.01 vs. NASH group (*n* = 10). Figures are prepared by GraphPad Prism 6.0 310 and one-way ANOVA with post hoc Tukey’s multiple comparisons tests were performed with SPSS 19.0. HSL, hormone-sensitive lipase; NASH, nonalcoholic steatohepatitis; PKA, protein kinase A.

Basically, the body weight of Ctrl and NASH group mice were not different before NC or HFD feeding (Fig. 1*B*). In comparison with Ctrl group, NASH mice were characterized by higher monthly absolute and percent changes in body weight, higher final body weight, and higher adiposity (high-fat mass and VAT weight; Fig. 1, *B–D*). Before ALZET/PSB0777 administration, although lower body weight was observed at two time points in NASH + PSB0777 group than that in NASH group, the monthly percent changes in body weight at different timepoints were not different between NASH and NASH + PSB0777 groups. Meanwhile, significant lower A_2A_R (*ADORA2A*) expression was associated with lower adenylyl cyclase (AC), cAMP, and PKA expressions in VAT of NASH group compared with the expressions of these markers in VAT of Ctrl group mice (Fig. 1, *E–I*, Fig. 2, *C–F*, and Supplemental Fig. S9). In line with the increased triglyceride content/lipogenesis [% of ORO stained (+) area] in VAT of NASH group, significantly lower *HSL* (lipolytic marker) and higher *SREBP1c/FASN* (lipogenic marker) levels in NASH group compared with those Ctrl group (Fig. 1, *K–L* and Fig. 2, *A* and *B*) were found.

After the restoration of A_2A_R expression in VAT by chronic PSB0777 treatment, in NASH + PSB0777 group, the normalization of A_2A_R-AC-cAMP-PKA cascades was accompanied by the decrease in triglyceride content/lipogenesis and fat mass were observed (Fig. 1, *E–L*, Fig. 2, *C–F*, and Supplemental Fig. S9). In the liver of NASH + PSB0777 group, similar trends of BSP0777-related restoration of AC-cAMP-PKA cascades and suppression of lipogenesis, as observed in VAT were found (Figs. 1 and 2, *A–F*, and Supplemental Fig. S9). These results indicated that paralleled pathogenic changes in VAT and livers in NASH mice can be simultaneously suppressed by chronic PSB0777 treatment.

### The Suppression of Inflammation by PSB0777 Treatment in VAT of NASH Mice Were Accompanied by Improvement in Liver

In comparison with Ctrl group, the significant downregulation of A_2A_R-AC-cAMP-PKA cascades was associated with the upregulation of inflammatory (ALT, MCP-1, and CD68) markers and the increase in the number of infiltrated CD11b^ + ^F4/80^+^ cells in VAT and liver of NASH mice (Fig. 2, *C–J* and Supplemental Figs. S1–S4 and S9). Notably, the restoration of the expression of AC-cAMP-PKA cascades by chronic A_2A_R activation simultaneously suppressed inflammatory markers and inflammation in VAT and liver of NASH + PSB0777 mice. However, the inflammatory markers and inflammation in VAT and liver of Ctrl group were not significantly different from those of Ctrl + PSB0777 group.

### Coincubation with A_2A_R Agonist Suppressed the Palmitic Acid-like Changes Induced by CMnash in Cell Lysates of AML-12 Cells

**Figure 3.**
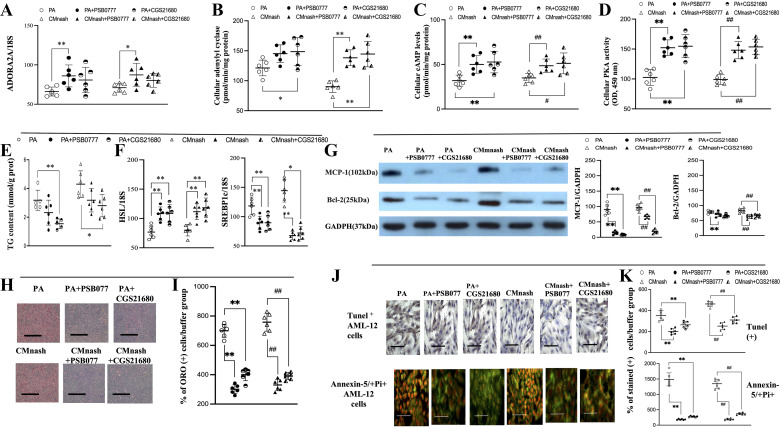
Coincubation with A_2A_R agonist (PSB0777 or CGS21680) suppressed the palmitic acid (PA, 0.2 mM)-like lipotoxic effects induced by CMnash in the AML-12 cell lysates. Relative (%/buffer group) mRNA (/18S) and protein (/GADPH) levels of ADORA2A, MCP-1, and Bcl-2 (*A* and *G*); adenylyl cyclase levels (*B*), cAMP levels (*C*), and PKA activity in cell lysates (*D*); TG content (mmol/g prot) and HSL and SREBP1c levels in cell lysates (*E* and *F*); representative image (×200, scale bar 100 μm) and scatter dot plot of relative (%/buffer group) Oil red O stained (+) area of AML-12 cells (×200, bar scale, 100 μm) (*H* and *I*); representative image and scatter dot plot of Tunel+ and Annexin-5+Pi+ cells (×200, scale bar 100 μm) among different pretreated AML-12 cells (×630, bar scale, 25 μm) (*J* and *K*). CMnash: conditioned media from VAT of NASH mice (*n* = 10). *,**P < 0.05, 0.01 vs. PA group; #,##*P* < 0.05, 0.01 vs. CMnash group. Figures are prepared by GraphPad Prism 6.0 310 (GraphPad Software, La Jolla, CA) and *t* test between PA/CMnash vs. or PA+PSB0777/CMnash+PSB0777 or PA/CMnash vs. or PA+CGS21680/CMnash+PSB0777 were performed with SPSS 19.0 (SPSS, Inc., Chicago, IL). HSL, hormone-sensitive lipase; NASH, nonalcoholic steatohepatitis; PA, palmitic acid; PKA, protein kinase A; TG, triglyceride; VAT, visceral adipose tissue.

Notably, the conditioned media (CM) of VAT of NASH mice (CMnash) trigger palmitic acid (PA)-like lipotoxic [the upregulation of inflammatory (MCP-1), lipogenic (SREBP1c/FASN), and apoptotic (Bcl-2, Tunel+, and annexin-5^+^/Pi^+^) markers] in AML-12 cell systems (Fig. 3 and Supplemental Fig. S10). The similarity of the in vitro effects of CMnash and palmitic acid (PA) in several experiments suggests that the PA content of the CMnash may be the operative factor for its lipotoxic effects.

The baseline levels of adenylyl cyclase, cAMP, and PKA activity in buffer only group of AML-12 cells were 178 ± 5 (pmol/min/mg protein), 88 ± 4 (pmol/mg protein), and 239 ± 13 (OD, 450 nm), separately. Notably, the abovementioned in vitro lipotoxic effects triggered by PA and CMnash were accompanied by the downregulation of ADORA2A, adenylyl cyclase, cAMP, PKA, and lipolytic (HSL) marker expressions in cell lysates of AML-12 cells (Fig. 3). Meanwhile, CMnash induced the lipogenesis (increased intracellular TG levels) in AML-12 cells like the PA-induced effects (Fig. 3, *H–I*).

Significantly, the coincubation with A_2A_R agonist (PSB0777) attenuated the PA or CMnash-related effects on various lipotoxic markers in cell lysates of AML-12 and lipogenesis of AML-12 cells (Fig. 3 and Supplemental Fig. S10). In addition to PSB0777, another A_2A_R agonist CGS21680 also induced the attenuation of PA and CMnash-induced pathogenic signals. These results further suggested that CMnash-released substance with lipotoxic effects that can be abolished by activation of A_2A_R agonist in AML-12 cell systems (Fig. 3).

### Paralleled Beneficial Effects of PSB0777 Treatment in VAT and Heart of NASH Mice

**Figure 4.**
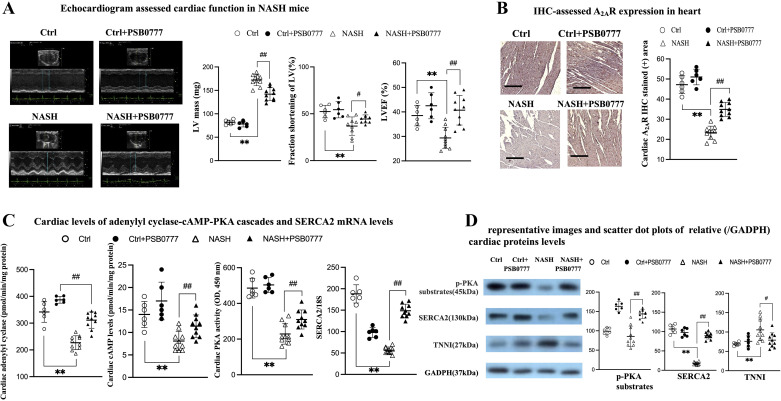
Beneficial effects of PSB0777 treatment in VAT of male NASH mice were accompanied by significant attenuation of cardiac dysfunction by normalization of cardiac A2AR-AC-cAMP-PKA cascades. Representative images and bar graph of cardiac function (LV mass, fraction of shortening of LV, and LVEF; *A*); representative images (×200, scale bar 100 μm) and scatter dot plot of IHC-assessed cardiac A2AR levels (*B*); cardiac levels and activity of adenylyl cyclase (AC), cAMP, PKA, SERCA2, and TNNI (*C* and *D*). **P < 0.01 vs. Ctrl group; #,##*P* < 0.05, 0.01 vs. NASH group. Figures are prepared by GraphPad Prism 6.0 310 and one-way ANOVA with post hoc Tukey’s multiple comparisons tests were performed with SPSS 19.0. IHC, immunohistochemical; NASH, nonalcoholic steatohepatitis; PA, palmitic acid; PKA, protein kinase A; VAT, visceral adipose tissue.

In comparison with Ctrl group, the significant low levels of cardiac A_2A_R-AC-cAMP-PKA expressions and the increase in the serum cardiac injury (CK and cTnI) markers were associated with remarkable cardiac dysfunction (increased LV mass, decreased fraction shortening of LV, and LVEF) in NASH mice with increased VAT mass and inflammation (Table 2 and Fig. 4*A*).

**Table 2.** Various serum markers among mice

	Ctrl (*n* = 6)	Ctrl+PSB0777 (*n* = 6)	NASH (*n* = 10)	NASH+PSB0777 (*n* = 10)
Serum MCP-1 level, pg/mL	83 ± 3	72 ± 3	150 ± 5**	96 ± 4##
Serum (alanine aminotransferase) (ALT, U/L)	51.3 ± 4.8	44.1 ± 8.6	115.9 ± 18.4**	89.5 ± 16.9#
Serum cardiac CK, U/L	102 ± 8	89 ± 3.5	238 ± 11**	195 ± 10.5##
Serum cardiac cTnI level, ng/mL	0.47 ± 0.02	0.39 ± 0.02	2.5 ± 0.1**	1.6 ± 0.12#

Values are means ± SE. **P < 0.01 vs. Ctrl group; #,##*P* < 0.05, 0.01 vs. NASH group.

**Figure 5.**
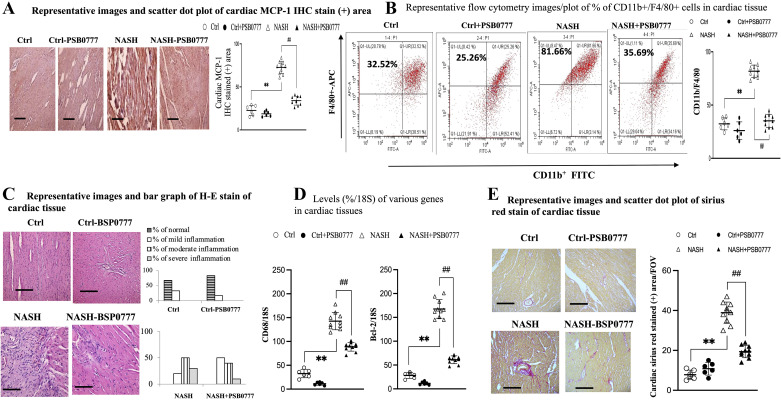
Chronic A_2A_R agonist treatment suppressed cardiac inflammatory, apoptotic, and fibrotic makers. IHC-assessed cardiac MCP-1 levels (×200, scale bar 100 μm; *A*); infiltration of CD11b+F4/80+ cells in homogenates of cardiac tissue among groups (*B*); H&E stained (×200, scale bar 100 μm) assessed cardiac inflammation among group (*C*). Cardiac PKA, inflammatory, apoptotic, and fibrotic markers [Degree of cardiac inflammation was classified as mild (mononuclear cells infiltration < 25% of the tissue), moderate (25%–50% of the tissue), or severe (> 50%) of the tissue section; *D*]; Sirius red stain-assessed cardiac fibrosis (×200, scale bar 100 μm; *E*). Figures are prepared by GraphPad Prism 6.0 310 and one-way ANOVA with post hoc Tukey’s multiple comparisons tests were performed with SPSS 19.0. **P < 0.01 vs. Ctrl group; ##*P* < 0.01 vs. NASH group. H&E, hematoxylin and eosin; IHC, immunohistochemical; PKA, protein kinase A.

Notably, NASH-related cardiac dysfunction was associated with high levels of cardiac inflammatory (increased MCP-1 and CD68, increased flow cytometry or H&E stained-assessed infiltrated inflammatory cells, Fig. 4, *B–D*, Fig. 5, *A–D*, and Supplemental Figs. S5–S8), cardiac injury (increased TNNI, Fig. 4*D* and Supplemental Fig. S10), cardiac apoptotic markers (increased Bcl-2, Fig. 5*D*), and cardiac fibrosis (increased Sirius red stained-positive area) in NASH mice. Sarco/endoplasmic reticulum Ca^2 + ^ATPase (SERCA2) plays a critical role in the contraction-relaxation cycle of the heart. In NASH mice heart, decreased expression of contraction (decreased SERCA2) markers (Fig. 4*D*) were observed.

In other words, downregulated A_2A_R-AC-cAMP-PKA signals-related paralleled cardiac and VAT dysfunction were observed in NASH mice (Figs. 1–2 and 4–5). Furthermore, chronic A_2A_R agonist PSB0777 treatment significantly reversed the abovementioned pathogenic signals and improved cardiac dysfunction in NASH mice (Table 2 and Fig. 4*A*).

### Coincubation with A_2A_R Agonist Suppressed the Palmitic Acid-like Changes Induced by CMnash in Cell Lysates of H92c Cells

**Figure 6.**
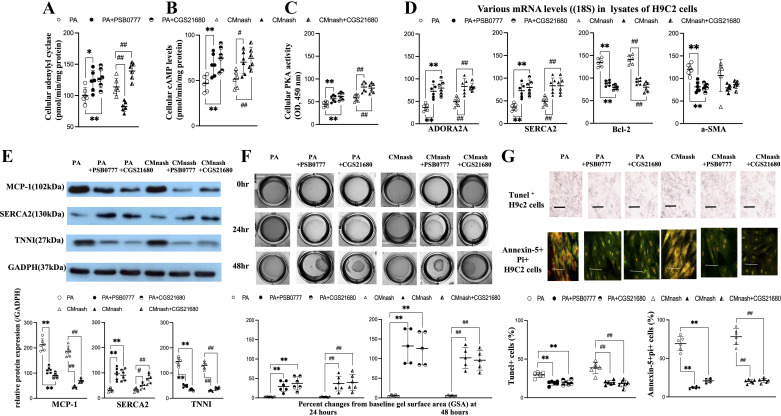
Coincubation with A_2A_R agonist (PSB0777 or CGS21680) suppressed the palmitic acid (PA, 0.2 mM)-like lipotoxic effects induced by CMnash in the H9c2 cell lysates. Levels of adenylyl cyclase, cAMP, and PKA activity in cell lysates (*A–C*); representative images and bar graph (/18S or GADPH) of the expression levels of ADORA2A, inflammatory (MCP-1), injury (TNNI), fibrotic (αSMA), apoptotic (Bcl-2), and contracting (SERCA2) makers in lysates of H9c2 cells (*D–E*); collagen gel contraction assay: relative percent changes of gel surface area (GSA) of monolayer of H9c2 (*F*); percentage of tunel+/annexin-5+Pi+ H9c2 cells after different treatments (×630, bar scale, 25 μm), *n* = 6 in each group (*G*). *,**P < 0.05, 0.01 vs. PA group; #,##*P* < 0.05, 0.01 vs. CMnash group. Figures are prepared by GraphPad Prism 6.0 310 and *t* test between PA/CMnash vs. or PA+PSB0777/CMnash+PSB0777 or PA/CMnash vs. or PA+CGS21680/CMnash+PSB0777 were performed with SPSS 19.0. PKA, protein kinase A.

Notably, the conditioned media (CM) of VAT of NASH mice (CMnash) trigger palmitic acid (PA)-like lipotoxic [increase in inflammatory (MCP-1)/injury (TNNI)/apoptotic (Bcl-2, Tunel^+^, and annexin-5^+^/Pi^+^)/fibrotic (αSMA)] effects in H9c2 cell systems. Particularly, abovementioned in vitro lipotoxic effects triggered by PA and CMnash were accompanied by the downregulation of ADORA2A, adenylyl cyclase, cAMP, PKA, and contraction (SERCA2) marker expressions in cell lysates of H9c2 cells (Fig. 6). Meanwhile, CMnash induced the hypocontraction of H9c2 cells like the PA-induced effects (Fig. 6 and Supplemental Fig. S11).

The baseline levels of adenylyl cyclase, cAMP, and PKA activity in buffer only group of H9c2 cells were 165 ± 7 (pmol/min/mg protein), 98 ± 13 (pmol/mg protein), and 221 ± 20 (OD, 450 nm), separately. Significantly, the coincubation with A_2A_R agonist (PSB0777) attenuated the PA or CMnash-related effects on various lipotoxic markers in cell lysates of H9c2 and hypocontraction/apoptosis of H9c2 cells (Fig. 6 and Supplemental Fig. S11). In addition to PSB0777, another A_2A_R agonist CGS21680 induced the attenuation of PA and CMnash-induced pathogenic signals. These results further suggested that CMnash-released substance with lipotoxic, proapoptotic, and anticontraction effects that can be abolished by activation of A_2A_R agonist in H9c2 cell systems.

## DISCUSSION

In NASH mice with systemic adiposity, downregulated A_2A_R-AC-cAMP-PKA signals and inflammation of visceral adipose tissue (VAT) were associated with hepatic/cardiac inflammation, apoptosis, fibrosis, and cardiac hypocontractility, all these abnormalities were reversed by chronic systemic activation of A_2A_R with A_2A_R agonist PSB0777.

The progressive increased production of inflammatory cytokines from inflamed VAT is observed as the disease progressed from NAFLD to NASH (34). In A_2A_R disruption in HFD-fed mice is associated with increased VAT inflammation and severity of NAFLD through significantly increasing the macrophage-derived proinflammatory mediator release (14, 15, 35). Especially, in HFD-induced NASH mice, high levels of MCP-1 derived from VAT and liver reaches the circulation and contribute to the disease progression (36). In the human airway smooth muscle cell system, the increase in intracellular cAMP decreases the MCP-1 expression and production (37). In HFD-fed obese mice, chronic A_2A_R agonist treatment increases cAMP levels, suppresses MCP-1 levels, and decreases macrophage infiltration in VAT (21, 38).

In concordance with the previous study (39), 24-wk HFD plus streptozotocin (STZ) injection successfully induces cardiac dysfunction in C57Bl/6 mice of our study. Enhancement of cardiac A_2A_R signals protect mice from pressure overload-induced cardiac fibrosis by suppression of inflammatory mediators released from cardiomyocytes (40).

cAMP/PKA pathways activating agents, such as GLP-1 agonists, are potential drugs to benefit both in NASH and cardiac dysfunction (41, 42). GLP-1 agonists can enhance insulin sensitivity, inhibit lipogenesis of hepatocyte isolated from NASH rats, and suppresses hepatic steatosis by increasing cAMP-PKA activity (43, 44). GLP-1 metabolite can prevent ischemic cardiac injury by increasing adenosine cyclase (AC)-dependent cAMP and activation of PKA in human coronary artery endothelial and smooth muscle cells (45).

In addition, cAMP can enhance the contractile function of cardiomyocytes through increase in the Ca^2+^ influx and subsequently triggers the release of intracellular stores of Ca^2+^ from the sarcoplasmic reticulum (SR) by activating sarcoplasmic reticulum (SR) Ca^2+^-ATPase (SERCA2) [SERCA2] expression. Activation of cardiac A2AR signals protects mice from pressure overload-induced cardiac hypocontractility by enhancing SERCA2-mediated increasing systolic intracellular calcium and rapid calcium reuptake from cardiomyocytes (40). In myocardial infarction (MI) rats, A2AR agonist treatment ameliorated the post-MI cardiac dysfunction by increasing SERCA2 expression (46–48). A2AR-AC-cAMP-PKA axis indicated that the activation of A2AR can stimulate adenosine cyclase (AC), which is a membrane effector synthesizing cAMP from ATP, to increase the cAMP formation (22). The cAMP-dependent protein kinase (PKA) signaling pathway is the primary means by which the heart regulates moment-to-moment changes in contractility and metabolism.

cAMP and its downstream PKA have anti-inflammatory effects by inhibiting the production of MCP-1. Cardiomyocyte-targeted expression of MCP-1 in mice results in increase myocardial apoptotic activity and ventricular dysfunction probably through persistent cytosolic Ca^2+^ influx (49–52). Enhancement of protein kinase A (PKA) activity increases calcium flux and increases cardiac muscle contractility by stimulating the functions of SERCA (53). Persistent increases in Ca^2+^ influx cause cardiac myocyte apoptosis if the excessive influx results in sarcoplasmic reticulum (SR) Ca^2+^ overload (50–52). Significantly, our study revealed that chronic A_2A_R treatment suppressed NASH-increased cardiac apoptotic activities and hypocontractility by inhibition of cardiac MCP-1 level and restoration of cardiac AC, cAMP, and SERCA2 expression. Perhaps the improvements in the cardiac dysfunction were due to A_2_AR agonist in the VAT, but it could also be that the improvements in the heart were due to direct action of A_2A_R on the heart itself. Culturing cardiomyocytes in conditioned media generated from the VAT of NASH mice with PSB0777 provide much stronger evidence that A_2_AR agonist in VAT leads to an altered secretome that exerts beneficial effects on heart cells.

The hormone-sensitive lipase (HSL) is a lipolytic enzyme that can be activated by the increased cAMP and cAMP-dependent PKA (54). HSL can release nonesterified fatty acids from adipose tissue. In Fig. 2*A*, lower HSL expression in NASH VAT than that in Ctrl VAT might be indicated that the release of FFA did not increase in VAT of NASH mice. Moreover, higher HSL expression was noted in NASH + PSB0777 VAT than NASH VAT. Notably, increased proinflammatory cytokines (CD68, MCP-1, infiltrated CD11b + F4/80+ cells) markers were observed in VAT of NASH compared with Ctrl group, whereas these are significantly suppressed in NASH + PSB077 group (Fig. 2, *C–I*). Possibly, proinflammatory cytokines from NASH VAT might be the main secretome to cause injury in AML-12 and cardiac H9c2 cells.

Similar to in vitro PA-induced effects in AML-12 and H9C2 cells system of current study, the conditioned media (CM) from VAT of NASH mice (CMnash)-induced the downregulation of A_2A_R-AC-cAMP-PKA axis, the upregulation of lipogenic/inflammatory/fibrotic signals, H9C2 apoptosis, the suppression of contraction signals and contractions of H9C2 cells. Furthermore, both PA and CMnash-induced effects were abolished by the coincubation with A_2A_R agonist PSB0777 or CGS21680. These in vitro results further support the important roles of VAT-released saturated fatty acid (SFA) such as PA and corresponding signals in the regulation of A_2A_R-AC-cAMP-PKA axis-related effects in liver and heart of NASH mice. However, validation in primary mouse hepatocytes or cardiomyocytes from healthy mice is necessary to strengthen the findings on the immortalized cells AML-12 or H9C2 cells in future studies.

PKA suppresses the hepatic lipogenesis through direct inhibition of sterol regulatory element-binding protein (SREBP) (55). cAMP and PKA activation attenuate SREBP-1 activity and SREBP-1-mediated adipogenesis in liver cell lines and obese db/db mice (56–57). Particularly, in addition to similar findings as previous study in VAT (58), the downregulation of A_2A_R-AC-cAMP-PKA axis was accompanied by increased SREBP1c/FASN markers and decreased lipolytic marker (HSL) in liver of NASH mice. Chronic A_2A_R agonist treatment, restored HSL activity, and suppressed SREBP1c/FASN level in VAT and liver, decrease the fat mass in NASH mice of the current study.

In conclusion, chronic A_2A_R agonist treatment is the potential agent to inhibit VAT inflammation-driven injury, steatosis, inflammation, apoptosis, fibrosis in liver and suppress hypocontractility signals in heart of NASH, and subsequently improve cardiac dysfunction in NASH mice.

## DATA AVAILABILITY

Data will be made available upon reasonable request.

## SUPPLEMENTAL DATA

10.6084/m9.figshare.24907584.v1 Supplemental Material and Methods, Supplemental Figs. S1–S11: https://doi.org/10.6084/m9.figshare.24907584.v1.

## GRANTS

This work was funded by Taipei Veterans General Hospital (Grant Nos.: V113C-024, VTA113-A-4-2&V113D71-001-MY2-1, CIC36, V113EA-005), Ministry of Science and Technology (Taiwan) (Grant Nos.: NSTC 112-2314-B-A049-043-MY3, MOST-110-2511-H-A49A-504-MY3, MOST 110-2314-B-001-003; MOST 111-2314-B-001-009), and National Yang-Ming Chiao Tung University (112Q58504Y).

## DISCLOSURES

No conflicts of interest, financial or otherwise, are declared by the authors.

## AUTHOR CONTRIBUTIONS

T.-L.L. and Y.-Y.Y. conceived and designed research; T.-L.L. performed experiments; C.-C.H., H.-Y.Y., and H.-C.L. analyzed data; H.-Y.Y., R.L., H.-C.S., and H.-C.L. interpreted results of experiments; C.-C.H., R.L., and H.-C.S. prepared figures; T.-L.L. edited and revised manuscript; Y.-Y.Y. approved final version of manuscript.
